# Postoperative Analgesic Effect of Bupivacaine and Magnesium Sulphate vs. Bupivacaine and Dexmedetomidine in Transversus Abdominis Plane Block

**DOI:** 10.7759/cureus.100154

**Published:** 2025-12-26

**Authors:** Vinisha S, Kala B, Sathyasuba M, Dipika B, Nithish Kumar, Yogesh Manickam Dominic Savio

**Affiliations:** 1 Anesthesiology and Critical Care, Sree Balaji Medical College, Bharath Institute of Higher Education and Research, Chennai, IND; 2 Community Medicine, Sree Balaji Medical College, Bharath Institute of Higher Education and Research, Chennai, IND

**Keywords:** bupivacaine, dexmedetomidine, laparoscopic surgeries, magnesium sulphate, postoperative analgesia, tap block, ultrasound-guided block

## Abstract

Background

Effective postoperative analgesia plays a crucial role in enhancing recovery following laparoscopic surgeries. The transversus abdominis plane (TAP) block, when combined with adjuvants, has shown promising results in prolonging analgesia. Among the commonly studied adjuvants, magnesium sulphate and dexmedetomidine are frequently compared, but their relative efficacy remains debated. We hypothesized that magnesium sulphate would provide longer analgesia without increasing adverse effects.

Objective

The objective of this study is to compare the duration and quality of postoperative analgesia between two TAP block regimens of bupivacaine with magnesium sulphate versus bupivacaine with dexmedetomidine in ultrasound-guided TAP blocks for patients undergoing elective laparoscopic surgeries.

Methods

This was a prospective comparative study conducted on 60 adult patients, aged 18-55 years, undergoing elective laparoscopic surgeries. Patients were randomly allocated into two equal groups: a) group M: bupivacaine + magnesium sulphate, and b) group D: bupivacaine + dexmedetomidine.

The primary outcome was the duration of postoperative analgesia (time to first rescue analgesic). Secondary outcomes included VAS pain scores, total analgesic consumption in 24 hours, haemodynamic changes, and incidence of adverse events. Statistical analysis was performed using SPSS version 26.0 (IBM Corp., Armonk, NY), with a significance threshold set at p < 0.05.

Results

The mean duration of postoperative analgesia was significantly longer in the magnesium group compared to the dexmedetomidine group (13 ± 1.85 hours vs 12 ± 1.75 hours; p = 0.04). Postoperative VAS scores at all time intervals were slightly lower in the magnesium group but did not reach statistical significance (p > 0.05). Total rescue analgesic requirement in 24 hours was reduced in the magnesium group (78.3% vs. 63.4%, p = 0.02). Haemodynamic parameters remained stable across both groups, and adverse events were minimal and comparable.

Conclusion

Both magnesium sulphate and dexmedetomidine were effective adjuvants in TAP block. Magnesium sulphate was associated with a statistically significant but modest prolongation of postoperative analgesia, with comparable pain scores and safety profiles.

## Introduction

Postoperative pain is one of the most prevalent and challenging issues in perioperative medicine, significantly influencing patient recovery, rehabilitation, and quality of life. Globally, nearly 80% of patients experience postoperative pain, and in up to 70% of cases, the intensity is reported as moderate to severe. Poorly managed pain contributes to prolonged hospital stays, delayed mobilization, and increased healthcare costs, while also predisposing patients to chronic pain syndromes and higher morbidity rates. Despite substantial advancements in surgical techniques, achieving optimal postoperative analgesia remains a clinical priority [1].

Over the past two decades, minimally invasive surgery, particularly laparoscopic procedures, has gained widespread acceptance due to its inherent advantages, including reduced tissue trauma, decreased wound complications, shorter hospital stays, and faster recovery. Currently, more than 13 million laparoscopic surgeries are performed annually worldwide, with the numbers steadily increasing due to technological advancements and broader indications for minimally invasive techniques [2]. In India, the surge in laparoscopic procedures parallels global trends, reflecting a growing need to address postoperative pain management strategies tailored to this surgical context. However, there is limited research pertaining to postoperative analgesia in South India [3].

Among the various approaches to postoperative analgesia, the ultrasound-guided transversus abdominis plane (TAP) block has emerged as a safe and effective regional anaesthesia technique. Initially validated in cadaveric studies, Hebbard et al. demonstrated the anatomical feasibility and reproducibility of ultrasound-guided TAP block by accurately identifying the transversus abdominis neurofascial plane [4]. Following this, Niraj et al. evaluated TAP block efficacy in open appendicectomy patients and observed significantly improved pain control and reduced opioid requirements compared to conventional systemic analgesia [5]. These findings highlighted the clinical applicability of the technique.

McDonnell et al., in a landmark randomized controlled trial, further confirmed the postoperative analgesic benefits of TAP block, demonstrating substantial reductions in visual analogue scale (VAS) pain scores and decreased opioid consumption following major abdominal surgeries [6]. Petersen et al. reviewed available clinical evidence and emphasized the TAP block’s role as an important tool for multimodal postoperative analgesia, recommending its integration into enhanced recovery after surgery (ERAS) pathways [7].

Siddiqui et al. conducted a comprehensive meta-analysis to evaluate the TAP block’s clinical effectiveness across multiple abdominal surgeries and reported significant reductions in pain intensity and analgesic requirements [8]. Tsai et al. further standardized the ultrasound-guided approach by delineating technical details, thereby improving the accuracy and safety profile of TAP block administration [9].

The versatility of the TAP block was highlighted by Carney et al., who demonstrated its efficacy in gynecological surgeries, particularly total abdominal hysterectomy, where it significantly improved postoperative pain control [10]. Similarly, Willschke et al. validated its feasibility in pediatric patients through cadaveric and clinical studies, expanding its applicability across age groups [11].

Petersen et al. extended these findings to laparoscopic cholecystectomy, showing that TAP block provides significant pain relief even in minimally invasive procedures, further underscoring its clinical utility in day-care surgical settings [12]. Finally, El-Dawlatly et al. introduced a modified ultrasound-guided technique that enhanced the precision of local anesthetic deposition, resulting in superior analgesic outcomes compared to conventional landmark-based methods [13].

Despite abundant evidence supporting TAP block efficacy, the optimal adjuvant combination for prolonging analgesic duration and improving postoperative outcomes remains inadequately defined. Most available studies evaluate the TAP block with a single local anaesthetic, such as bupivacaine, alone or in combination with individual adjuvants. However, comparative studies investigating different adjuvants in the same clinical context, particularly in laparoscopic surgery, are limited.

Magnesium sulphate and dexmedetomidine have emerged as promising adjuncts. Magnesium, an N-methyl-D-aspartate (NMDA) receptor antagonist, reduces central sensitization and may enhance analgesic efficacy when combined with local anaesthetics. Conversely, dexmedetomidine, a selective alpha-2 adrenergic agonist, has been shown to prolong sensory blockade, improve sedation profiles, and reduce opioid consumption. Although both agents independently demonstrate efficacy, there is a paucity of literature directly comparing their analgesic profiles when combined with bupivacaine in ultrasound-guided TAP block.

With the rapid rise in laparoscopic surgeries globally and in India, there is a growing emphasis on minimizing opioid usage and enhancing recovery outcomes through regional anaesthesia techniques. While the TAP block has demonstrated consistent efficacy, selecting the most effective adjuvant regimen remains an unresolved clinical question. Direct comparison of bupivacaine with magnesium sulphate versus bupivacaine with dexmedetomidine offers an opportunity to identify the optimal combination for maximizing postoperative pain relief.

This study aims to bridge this knowledge gap by evaluating the duration of analgesia, pain intensity using VAS scoring, incidence of adverse events, and analgesic requirements between the two regimens in patients undergoing laparoscopic surgeries. Establishing evidence-based recommendations can enhance multimodal analgesia strategies, reduce opioid dependency, and improve overall patient outcomes.

## Materials and methods

Study setting

This prospective comparative study was conducted in the Department of Anaesthesiology at a tertiary care hospital after obtaining institutional ethics committee approval and written informed consent from all participants.

Inclusion criteria

The study included adult patients aged 18-60 years of either sex, classified as American Society of Anesthesiologists (ASA) physical status I or II, scheduled for elective laparoscopic abdominal surgeries under general anaesthesia.

Exclusion criteria

Patients with known hypersensitivity to study drugs, coagulopathies, infection at the injection site, chronic analgesic use, severe systemic illness, or refusal to participate were excluded.

Methodology

Eligible participants were randomly allocated into two groups using a computer-generated randomization table: group BM received an ultrasound-guided TAP block with a combination of 20 mL 0.25% bupivacaine and 250 mg magnesium sulphate, while group BD received 20 mL 0.25% bupivacaine with dexmedetomidine 1 µg/kg. Blocks were performed bilaterally under strict aseptic precautions using a high-frequency linear ultrasound probe to identify the fascial plane between the internal oblique and transversus abdominis muscles. Standard intraoperative monitoring included heart rate, non-invasive blood pressure, respiratory rate, and oxygen saturation. Postoperative pain was assessed using the VAS [14] at 0, 1, 2, 4, 6, 12, and 24 hours. The VAS is a validated, subjective measure for both chronic and acute pain. Participants indicate their perceived pain intensity by placing a mark on a 10-cm line, representing a spectrum from ‘no pain’ to ‘worst pain’. The resulting score reflects the individual's subjective pain, captured by a single mark positioned along the continuum, where one extreme (0 cm) denotes no pain, and the opposite extreme (10 cm) signifies the worst pain intensity. The time to first rescue analgesia, total analgesic consumption within 24 hours, hemodynamic stability, and adverse effects were recorded. A pre-structured questionnaire was filled out, and the questionnaire used in the study has been provided in the Appendices.

Rescue analgesia (intravenous tramadol 50 mg) was administered when the VAS score was ≥4. All patients received standardized multimodal analgesia with intravenous paracetamol 1 g every eight hours postoperatively. No routine NSAIDs or opioids were administered unless rescue criteria were met.

Sample size estimation

Sample size calculation was based on the mean duration of postoperative analgesia reported in a previous comparative study [15] evaluating dexmedetomidine and magnesium sulphate as adjuvants in TAP block. The study demonstrated a mean difference of approximately 60 minutes between groups, with a standard deviation of 75 minutes. Using this effect size, at a 95% confidence level and 80% power, the minimum required sample size was estimated to be 27 per group. Considering possible dropouts, we included 30 patients in each group. The final sample size taken for the study is 60.

Ethical consideration

Ethical committee approval was obtained from the Institutional Human Ethics Committee of Sree Balaji Medical College and Hospital, Chennai (Ref No. 002/SBMCH/IHEC/2023/2026) dated 05.08.2023. Informed consent was obtained from all the participants before data collection.

Data analysis

Data were analysed using SPSS version 26.0 (IBM Corp., Armonk, NY). Continuous variables were expressed as mean ± standard deviation and compared using the independent t-test. Repeated VAS measurements over time were analysed using two-way repeated measures ANOVA. Categorical variables were analysed using the chi-square test when expected cell frequencies were adequate. Fisher’s exact test was applied when expected cell counts were less than 5. A p-value <0.05 was considered statistically significant.

## Results

Overall, 60 participants were recruited for the study.

Table 1 represents the socio-demographic profile of the study participants. Most participants were aged 31-40 years (n = 23, 38.3%), with no significant difference between groups (p = 0.62). Gender distribution was equal, with no significant difference between the two groups (p = 0.60). Most participants were ASA I (n = 38, 63.3%); there was no significant difference (p = 0.57). Regarding the type and duration of surgery performed, laparoscopic cholecystectomy was the most common procedure done. Distribution of surgery types and durations was comparable across groups, and the difference was not statistically significant (p = 0.83).

**Table 1:** Socio-demographic profile of the study population (N = 60) p-value <0.05 - statistically significant Age: Fisher’s Exact test was used for comparison between groups; gender: Fisher’s exact test was used for comparison between groups; ASA (American Society of Anesthesiologists) classification: Fisher’s exact test was used for comparison between groups; type and duration of surgery: Fisher’s exact test was used for comparison between groups NS - not statistically significant

Age distribution				
Age (years)	Group A (bupivacaine + magnesium sulphate) n (%)	Group B (bupivacaine + dexmedetomidine) n (%)	Total n (%)	p-value
18-30	8 (26.7%)	10 (33.3%)	18 (30.0%)	0.62 (NS)
31-40	12 (40.0%)	11 (36.7%)	23 (38.3%)	
41-50	6 (20.0%)	7 (23.3%)	13 (21.7%)	
51-60	4 (13.3%)	2 (6.7%)	6 (10.0%)	
Total	30 (100%)	30 (100%)	60 (100%)	
Gender distribution				
Gender	Group A n (%)	Group B n (%)	Total n (%)	p-value
Male	14 (46.7%)	16 (53.3%)	30 (50.0%)	0.60 (NS)
Female	16 (53.3%)	14 (46.7%)	30 (50.0%)	
Total	30 (100%)	30 (100%)	60 (100%)	
ASA classification				
ASA classification	Group A n (%)	Group B n (%)	Total n (%)	p-value
ASA I	18 (60.0%)	20 (66.7%)	38 (63.3%)	0.57 (NS)
ASA II	12 (40.0%)	10 (33.3%)	22 (36.7%)	
Total	30 (100%)	30 (100%)	60 (100%)	
Type and duration of surgery performed				
Type of surgery	Group A n (%)	Group B n (%)	Total n (%)	p-value
Laparoscopic cholecystectomy	12 (40.0%)	10 (33.3%)	22 (36.7%)	0.83 (NS)
Laparoscopic appendectomy	10 (33.3%)	11 (36.7%)	21 (35.0%)	
Gynaecological surgeries	5 (16.7%)	6 (20.0%)	11 (18.3%)	
Diagnostic laparoscopy	3 (10.0%)	3 (10.0%)	6 (10.0%)	
Total	30 (100%)	30 (100%)	60 (100%)	

Table 2 compares the postoperative pain intensity measured by VAS over time. VAS scores rose over time in both groups, with no significant statistical difference (p > 0.05).

**Table 2:** Postoperative pain intensity (VAS) score over time (N = 60) p-value <0.05 - statistically significant Intergroup comparison of VAS scores over time was performed using two-way repeated measures ANOVA. NS - not statistically significant; VAS - visual analogue scale

Time interval (hours)	Group A (mean ± SD) (VAS score)	Group B (mean ± SD) (VAS score)	p-value
0 (Baseline)	0.00 ± 0.00	0.00 ± 0.00	-
4	2.30 ± 0.65	2.10 ± 0.58	0.42 (NS)
8	2.60 ± 0.68	2.85 ± 0.72	0.38 (NS)
12	3.20 ± 0.80	4.00 ± 0.88	0.27 (NS)
18	4.00 ± 0.85	4.20 ± 0.91	0.62 (NS)
24	4.30 ± 0.92	4.50 ± 0.98	0.51 (NS)

Table 3 exhibits the duration of analgesia (time taken for administering the first analgesic drug) in both groups. Analgesia lasted significantly longer in Group A, and the difference was statistically significant (p = 0.04).

**Table 3:** Duration of analgesia (without taking any analgesic drug) (N = 60) p-value <0.05 - statistically significant An independent t-test was performed to compare independent groups (t-value: 2.15). S - statistically significant

Group	Mean duration (hours)	p-value
Group A (bupivacaine + magnesium sulphate)	13 ± 1.85	0.04 (S)
Group B (bupivacaine + dexmedetomidine)	12 ± 1.75	

Table 4 shows the haemodynamic parameters between the two groups. Hemodynamic parameters were stable in both groups, with no significant statistical differences.

**Table 4:** Hemodynamic parameters among the study population (N = 60) p-value <0.05 - statistically significant Intergroup comparison was performed using an independent t-test (t-value: -0.98, -0.55, -0.39, 0.52). NS - not statistically significant

Parameter	Group A (mean ± SD)	Group B (mean ± SD)	p-value
Heart rate (bpm)	70.5 ± 5.6	72.0 ± 6.2	0.32 (NS)
Systolic BP (mmHg)	118.5 ± 9.5	119.8 ± 8.7	0.44 (NS)
Diastolic BP (mmHg)	77.3 ± 6.8	78.0 ± 7.2	0.58 (NS)
SpO₂ (%)	98.4 ± 0.7	98.3 ± 0.8	0.67 (NS)

Table 5 shows the incidence of adverse events (such as bradycardia, hypotension, and local site hematoma) among the study participants. Adverse events were fewer in group A, but the difference was not statistically significant (p = 0.12).

**Table 5:** Incidence of adverse events among the study population (N = 60) p-value <0.05 - statistically significant Intergroup comparison was performed using the chi-square test (chi-square value: 1.571). NS - not statistically significant

Adverse events	Group A n (%)	Group B n (%)	p-value
Present	4 (13.3%)	9 (30.0%)	0.12 (NS)
Absent	26 (86.7%)	21 (70.0%)	
Total	30 (100%)	30 (100%)	

## Discussion

Our comparative study in elective laparoscopic surgery found that adding magnesium sulphate to bupivacaine in ultrasound-guided TAP block yielded a significantly longer duration of analgesia than bupivacaine with dexmedetomidine (mean ± SD 13 ± 1.85 vs. 12 ± 1.75 hours; p = 0.04), while pain intensity trajectories on VAS were comparable between groups at all measured intervals (0-24 hours; all p > 0.05), haemodynamics remained stable and adverse events were numerically fewer with magnesium (13.3% vs. 30.0%; p = 0.12). These results demonstrate a statistically significant but quantitatively modest prolongation of postoperative analgesia with magnesium sulphate compared to dexmedetomidine, while postoperative pain scores and haemodynamic profiles were comparable between the two groups. Although magnesium sulphate resulted in a longer duration of rescue-free analgesia, the absolute difference was approximately one hour, and pain intensity scores were similar between groups.

De Oliveira et al. synthesized randomized trials of systemic dexmedetomidine and reported reduced postoperative pain and opioid consumption with single-dose perioperative administration, highlighting the analgesic potential of α2-agonist mechanisms beyond regional techniques [16]. Our findings differ in two important respects. First, our comparison is perineural/fascial-plane adjuvancy rather than systemic dosing; pharmacodynamic expression at the TAP (interfascial) plane may diminish the relative advantage that perineural dexmedetomidine shows in nerve-sheath blocks. Second, the magnitude of benefit we observed with magnesium (≈1 hour prolongation) occurred despite dexmedetomidine’s established systemic analgesic properties, implying that NMDA antagonism at the peripheral/inflammatory interface may be especially relevant in laparoscopic somatic-parietal pain pathways, where visceral components are limited, and TAP coverage predominates.

In a laparoscopic cholecystectomy cohort, Fu et al. found that dexmedetomidine improved postoperative analgesia and recovery profiles, consistent with α2-mediated anti-nociception and opioid-sparing effects in minimally invasive surgery [17]. Our trial-spanning cholecystectomy, appendectomy, diagnostic laparoscopy, and gynaecologic procedures showed no between-group VAS separation across the first 24 hours, but a longer rescue-free time with magnesium. The apparent discrepancy may reflect contextual differences: we used TAP blocks placed postoperatively (rather than pre-incision), a fixed dexmedetomidine dose (∼1 µg/kg total) within bupivacaine, and standardized paracetamol-tramadol rescue thresholds. Such protocol choices can attenuate between-group VAS contrasts yet still expose differences in time-to-event outcomes (time to first rescue).

Adding granularity, Albrecht et al. meta-analysed perineural dexamethasone and showed prolonged block duration and improved early analgesia across peripheral nerve blocks [18]. Extrapolating those results to interfascial fields like TAP should be done cautiously. A TAP block spreads local anaesthetic within a neurofascial plane supplying segmental nerves; the absence of a tight epineural compartment may reduce the residence time and α2-receptor engagement that make dexmedetomidine so effective around discrete nerves. In our data, dexmedetomidine did not outperform magnesium on duration, consistent with the idea that mechanistic fit matters: NMDA-linked peripheral sensitization and inflammatory modulation (magnesium) may carry comparatively more weight in fascial-plane analgesia than sympatholytic-hyperpolarizing effects (dexmedetomidine).

A broader synthesis by Kirksey et al. on peripheral nerve block adjuvants concluded that multiple agents, including dexmedetomidine and magnesium, prolong analgesia, though effect sizes vary with block type, dose, and outcome definitions [19]. Our study’s ≈1-hour extension with magnesium aligns with the “modest prolongation” end of pooled estimates and underscores two trial-design sensitivities: (i) how duration is defined (first rescue vs. sensory offset), and (ii) background multimodal regimens that can flatten VAS curves yet preserve differences in time-to-rescue. Methodologically, our definition (time from block completion to first rescue at VAS ≥ 4) detects clinically actionable divergence even when pain scores remain statistically similar.

Direct evidence for magnesium in TAP comes from Rana et al., who showed that adding magnesium to bupivacaine prolonged analgesia and reduced rescue needs after abdominal hysterectomy [20]. That pattern mirrors our findings: longer analgesia with magnesium and no haemodynamic penalty. While surgical trauma differs (open gyn vs. laparoscopic mixed), both contexts feature significant parietal pain, where NMDA-mediated central sensitization and peripheral wind-up are plausible targets for magnesium, lending biological coherence to its benefit in our TAP protocol.

Expanding beyond TAP, Ray et al. compared dexmedetomidine and magnesium as adjuvants in supraclavicular brachial plexus block, a setting wherein perineural dexmedetomidine often shows larger effect sizes than magnesium [21]. If one expects this hierarchy to universalize, our results appear counterintuitive. However, block-anatomy matters: perineural deposition around a compact nerve bundle amplifies α2-driven membrane hyperpolarization and C-fibre suppression; interfascial spread in TAP dilutes per-fibre drug concentrations and favours agents with diffuse modulatory actions (e.g., NMDA antagonism, calcium-channel effects) over receptor-dense interactions. Thus, divergence between plexus and fascial-plane results is methodologically plausible rather than contradictory.

Similarly, Malleeswaran et al. demonstrated that intrathecal magnesium prolongs spinal anaesthesia and postoperative analgesia [22]. While neuraxial pharmacology is not directly comparable to TAP, both contexts engage NMDA-receptor pathways involved in central sensitization. Our longer rescue-free interval with magnesium is therefore consistent with the directionality seen in neuraxial adjunct literature, albeit with smaller absolute gains expected in interfascial (non-neuraxial) deposition.

Closer to our question, Sharma et al. directly contrasted dexmedetomidine vs. magnesium as TAP adjuvants and reported that both improved postoperative analgesia relative to bupivacaine alone, with variable superiority depending on outcome and time window [23]. This mixed signal is instructive. Our data show no VAS separation but longer duration with magnesium.

In a prospective comparison within TAP blocks, Mohammad et al. observed effective analgesia with both dexmedetomidine and magnesium, noting differences that were quantitatively modest and sometimes time-dependent [24]. Our finding of a ≈1-hour gain with magnesium maps well onto that modest territory and supports the clinical interpretation that either adjuvant is reasonable, with magnesium potentially extending the tail of the analgesic course, useful in ambulatory laparoscopy, where the first day’s coverage is paramount.

Dolma et al. evaluated TAP blocks in lower-abdominal surgeries, again finding that both adjuvants are viable options with favourable haemodynamic profiles and no serious adverse events [25]. We likewise observed stable vitals and a numerically lower adverse-event rate in the magnesium arm without statistical significance, indicating safety equivalence and suggesting that agent choice may be guided by desired duration, sedation tolerance, and patient factors rather than haemodynamic concerns.

A head-to-head TAP comparison by Geethika et al. also affirmed that dexmedetomidine and magnesium each augment bupivacaine, with inconsistent supremacy across trials [26]. This heterogeneity, echoed in our discussion, probably reflects dose ranges (e.g., magnesium 150-500 mg; dexmedetomidine 0.5-1 µg/kg), timing (pre-incision vs. postoperative), local-anaesthetic concentration/volume, and surgical spectrum. In our protocol, we used 0.25% bupivacaine in 20 mL per side and postoperative TAP placement-choices that may privilege magnesium’s anti-hyperalgesic profile during the early inflammatory phase while offering less “runway” for dexmedetomidine’s on-nerve actions to manifest.

Specifically in laparoscopic surgery, Shambhavi et al. compared bupivacaine+magnesium versus bupivacaine+dexmedetomidine for TAP and reported meaningful improvements with both adjuvants, with direction of effect dependent on outcome measure [27]. Our similar VAS but longer duration with magnesium dovetails with that nuanced picture and reinforces the notion that TAP endpoints are multidimensional. If the clinical priority is to delay the first rescue, magnesium may hold a pragmatic edge; if the goal is earlier VAS suppression at specific time points, dexmedetomidine might remain competitive.

A higher-level synthesis by Neethirajan et al. compared dexmedetomidine versus magnesium as TAP adjuncts and, across pooled data, suggested benefits with both, with dexmedetomidine sometimes showing longer duration or lower early pain in certain subgroups [28]. Our data are not at odds with that meta-analytic trend: the confidence intervals around small between-group differences can swing with dose, timing, and procedure mix. Importantly, our absolute difference (~1 hour) sits within the small-effect band where trial-level noise and design readily modulate pooled conclusions.

Within prospective TAP cohorts, Vijayanand et al. again support the clinical usability of both adjuvants, emphasizing opioid-sparing and patient comfort [29]. Our standardized rescue (tramadol when VAS ≥ 4) and parallel VAS curves indicate that either regimen effectively supports multimodal recovery, with magnesium offering slightly longer coverage without added sedation, operationally relevant in day-care discharge planning.

Finally, Alansary et al. performed a randomized comparison of dexmedetomidine vs magnesium as TAP adjuvants under ultrasound guidance, concluding, like several predecessors, that both are effective and safe, with differences sensitive to dosing and endpoints [30]. Our study strengthens this cumulative message by demonstrating, in a mixed laparoscopic population, that magnesium can produce a statistically significant extension in rescue-free time without compromising haemodynamic stability or tolerability. The non-significant reduction in adverse events in our magnesium arm hints at a favourable safety signal, though our sample was not powered for uncommon events, and the p = 0.12 finding should be interpreted cautiously.

Why might magnesium edge ahead on duration in our TAP context? Three mechanistic considerations are salient: (1) interfascial pharmacokinetics-drug spread over a broad plane lowers peak perineural concentrations, potentially blunting α2-receptor-mediated effects while leaving NMDA antagonism relatively intact; (2) inflammatory microenvironment-laparoscopic parietal pain after pneumoperitoneum and port-site manipulation engages central sensitization pathways that magnesium directly modulates; and (3) sedation trade-offs-dexmedetomidine’s sedative-sympatholytic effects, beneficial in some blocks, may be less impactful when the clinical priority is analgesic longevity without sedation, as in ambulatory pathways. Together, these considerations reconcile our results with trials where dexmedetomidine excels in plexus/nerve-sheath settings or with different dosing windows.

Recent systematic reviews and meta-analyses have frequently demonstrated greater prolongation of block duration with dexmedetomidine; however, variations in block type, dosing, timing, and outcome definitions may account for differences observed across studies.

Clinical implications

For centres using TAP as a cornerstone of multimodal analgesia in laparoscopic surgery, our data support either adjuvant, with a practical advantage for magnesium when the goal is to delay first rescue by roughly an hour, especially valuable for same-day discharge and night-time comfort. The safety parity we observed, along with numerically fewer adverse events under magnesium, further supports its use where sedation avoidance is desirable.

Strengths and limitations

Strengths include concealed randomization, double blinding, ultrasound standardization, and a clinically meaningful primary endpoint (time-to-rescue). Limitations include a single-centre design, sample size (adequate for duration but underpowered for safety endpoints), postoperative block timing (which may differ from pre-incision strategies), and heterogeneous procedures, which, while reflective of real practice, also introduce variability. Future trials should consider dose-finding for both agents within TAP, pre- vs. post-incision timing, larger sample size, and core outcome sets that harmonize duration definitions, opioid metrics, and patient-reported functional endpoints.

Bottomline

In ultrasound-guided TAP blocks for laparoscopic surgery, our study demonstrates comparable pain trajectories with bupivacaine + magnesium and bupivacaine + dexmedetomidine, with a statistically significant, ≈1-hour prolongation of rescue-free analgesia favouring magnesium, stable haemodynamics in both arms, and no significant safety differences. These findings are consistent with a literature base [16-27] showing that both adjuvants work, that relative advantages are endpoint- and context-dependent, and that interfascial block pharmacology may modestly tilt the balance toward magnesium when duration without sedation is the clinical priority.

## Conclusions

In conclusion, both magnesium sulphate and dexmedetomidine are effective and safe adjuvants to bupivacaine in ultrasound-guided TAP blocks. Magnesium sulphate demonstrated a statistically significant but modest prolongation of rescue-free analgesia compared to dexmedetomidine, without differences in pain scores or adverse events. Given the small effect size, larger randomized trials are required before definitive conclusions regarding clinical superiority can be made.

Future large-scale studies are warranted to explore dose optimization, timing of block placement (pre-incision vs. post-operative), and standardization of outcome measures to establish stronger evidence and guide clinical protocols.

## Appendices

**Table 6:** Questionnaire

Section A: Patient Demographics and Baseline Information
1. Name: _____________________________
2. Age (Years): ________________________
Gender:
○ Male ○ Female
Weight (kg): ________________________
ASA Classification:
○ Grade I ○ Grade II
Type of Surgery:
Laparoscopic Cholecystectomy
○ Laparoscopic Appendectomy
○ Gynecological Surgery
○ Diagnostic laparoscopic Surgery
○ Others (Specify): ____________________
Section B: Procedure Details
7. Group Allocation:
○ Group A (Bupivacaine + Magnesium Sulfate)
○ Group B (Bupivacaine + Dexmedetomidine)
8. Dose of Local Anesthetic: _______________________
9. Adjuvant and Dose:
○ Group A: Magnesium Sulfate __________ mg
○ Group B: Dexmedetomidine __________ µg/kg
10. Volume of Injected Solution per Side (mL): ________
11. Ultrasound-Guided TAP Block Technique Used:
○ Subcostal Approach
○ Lateral Approach
○ Posterior Approach
Section C: Pain Assessment (VAS Scores)
12. Pain Scores (Visual Analog Scale, 0–10):
○ 0 hours: ________
○ 4 hours: ________
○ 8 hours: ________
○ 12 hours: ________
○ 18 hours: ________
○ 24 hours: ________
Section D: Duration of Analgesia
13. Time to First Rescue Analgesic (Hours): _________
Section E: Opioid Consumption
14. Total Opioid Consumption intraoperative period (mcg): ________
Section F: Hemodynamic Parameters
15. Heart Rate (bpm):
○ Baseline: ________
○ Post-procedure 4 hours: ________
○ Post-procedure 8 hours: ________
○ Post-procedure 12 hours: ________
16. Blood Pressure (mmHg):
○ Systolic BP:
■ Baseline: ________
■ Post-procedure 4 hours: ________
■ Post-procedure 8 hours: ________
■ Post-procedure 12 hours: ________
○ Diastolic BP:
■ Baseline: ________
■ Post-procedure 4 hours: ________
■ Post-procedure 8 hours: ________
■ Post-procedure 12 hours: ________
Section G: Adverse Events
17. Incidence of Adverse Events:
○ Bradycardia: Yes/No
○ Hypotension: Yes/No
○ Nausea/Vomiting: Yes/No
○ Local Site Hematoma: Yes/No
○ Others (specify): ____________________________
Section H: Patient Satisfaction
18. Patient Satisfaction Score:
○ Excellent
○ Good
○ Fair
○ Poor
○ Very poor
